# Machine learning insight into the role of imaging and clinical variables for the prediction of obstructive coronary artery disease and revascularization: An exploratory analysis of the CONSERVE study

**DOI:** 10.1371/journal.pone.0233791

**Published:** 2020-06-25

**Authors:** Lohendran Baskaran, Xiaohan Ying, Zhuoran Xu, Subhi J. Al’Aref, Benjamin C. Lee, Sang-Eun Lee, Ibrahim Danad, Hyung-Bok Park, Ravi Bathina, Andrea Baggiano, Virginia Beltrama, Rodrigo Cerci, Eui-Young Choi, Jung-Hyun Choi, So-Yeon Choi, Jason Cole, Joon-Hyung Doh, Sang-Jin Ha, Ae-Young Her, Cezary Kepka, Jang-Young Kim, Jin-Won Kim, Sang-Wook Kim, Woong Kim, Yao Lu, Amit Kumar, Ran Heo, Ji Hyun Lee, Ji-min Sung, Uma Valeti, Daniele Andreini, Gianluca Pontone, Donghee Han, Todd C. Villines, Fay Lin, Hyuk-Jae Chang, James K. Min, Leslee J. Shaw

**Affiliations:** 1 Department of Radiology, New York-Presbyterian Hospital and Weill Cornell Medicine, New York, New York, United States of America; 2 Dalio Institute of Cardiovascular Imaging, Weill Cornell Medicine, New York, New York, United States of America; 3 Department of Cardiovascular Medicine, National Heart Centre, Singapore, Singapore; 4 Division of Cardiology, Severance Cardiovascular Hospital, Integrative Cardiovascular Imaging Center, Yonsei University College of Medicine, Seoul, South Korea; 5 VU Medical Center, Amsterdam, the Netherlands; 6 Myongji Hospital, Seonam University College of Medicine, Gyeonggi-do, South Korea; 7 CARE Hospital and FACTS Foundation, Hyderabad, India; 8 Centro Cardiologico Monzino, IRCCS, Milan, Italy; 9 Quanta Diagnostico Nuclear, Curitiba, Brazil; 10 Gangnam Severance Hospital, Seoul, South Korea; 11 Pusan National University Hospital, Busan, South Korea; 12 Ajou University Hospital, Gyeonggi-do, South Korea; 13 Cardiology Associates of Mobile, Mobile, Alabama, United States of America; 14 Inje University, Ilsan Paik Hospital, Gyeonggi-do, South Korea; 15 Gangneung Asan Hospital, Gangwon-do, South Korea; 16 Kangwon National University Hospital, Gangwon-do, South Korea; 17 Institute of Cardiology, Warsaw, Poland; 18 Wonju Severance Hospital, Gangwon-do, South Korea; 19 Korea University Guro Hospital, Seoul, South Korea; 20 Chung-Ang University Hospital, Seoul, South Korea; 21 Yeungnam University Hospital, Daegu, South Korea; 22 Asan Medical Center, University of Ulsan College of Medicine, Seoul, South Korea; 23 Severance Cardiovascular Hospital, Yonsei University Health System, Seoul, South Korea; 24 Department of Medicine, Stanford Medicine, Stanford, California, United States of America; 25 Department of Imaging, Cedars-Sinai Medical Center, Cedars-Sinai Heart Institute, Los Angeles, California, United States of America; 26 Department of Medicine, University of Virginia Health System, Charlottesville, Virginia, United States of America; 27 Cleerly, Inc, New York, New York, United States of America; Medizinische Universitat Graz, AUSTRIA

## Abstract

**Background:**

Machine learning (ML) is able to extract patterns and develop algorithms to construct data-driven models. We use ML models to gain insight into the relative importance of variables to predict obstructive coronary artery disease (CAD) using the Coronary Computed Tomographic Angiography for Selective Cardiac Catheterization (CONSERVE) study, as well as to compare prediction of obstructive CAD to the CAD consortium clinical score (CAD2). We further perform ML analysis to gain insight into the role of imaging and clinical variables for revascularization.

**Methods:**

For prediction of obstructive CAD, the entire ICA arm of the study, comprising 719 patients was used. For revascularization, 1,028 patients were randomized to invasive coronary angiography (ICA) or coronary computed tomographic angiography (CCTA). Data was randomly split into 80% training 20% test sets for building and validation. Models used extreme gradient boosting (XGBoost).

**Results:**

Mean age was 60.6 ± 11.5 years and 64.3% were female. For the prediction of obstructive CAD, the AUC was significantly higher for ML at 0.779 (95% CI: 0.672–0.886) than for CAD2 (0.696 [95% CI: 0.594–0.798]) (P = 0.01). BMI, age, and angina severity were the most important variables. For revascularization, the model obtained an overall area under the receiver-operation curve (AUC) of 0.958 (95% CI = 0.933–0.983). Performance did not differ whether the imaging parameters used were from ICA (AUC 0.947, 95% CI = 0.903–0.990) or CCTA (AUC 0.941, 95% CI = 0.895–0.988) (P = 0.90). The ML model obtained sensitivity and specificity of 89.2% and 92.9%, respectively. Number of vessels with ≥70% stenosis, maximum segment stenosis severity (SSS) and body mass index (BMI) were the most important variables. Exclusion of imaging variables resulted in performance deterioration, with an AUC of 0.705 (95% CI 0.614–0.795) (P <0.0001).

**Conclusions:**

For obstructive CAD, the ML model outperformed CAD2. BMI is an important variable, although currently not included in most scores. In this ML model, imaging variables were most associated with revascularization. Imaging modality did not influence model performance. Removal of imaging variables reduced model performance.

## Background

The evaluation of chest pain in patients with no prior known coronary artery disease (CAD) often includes invasive coronary angiography (ICA). However, the diagnostic yield of ICA in detecting obstructive coronary artery disease (CAD) can be as low as 23.0% to 40%. [1,2] This is partly a result of a broader patient selection criteria that includes lower risk patients, such as younger patients and those not having a prior positive stress test. This has led to the development of appropriate use criteria to guide ICA performance. [3] Even when adhering to this, obstructive CAD may be found in only 52.9% of patients with new onset stable chest pain and conversely, 30.9% of patients deemed inappropriate by these criteria have been found to have obstructive CAD. [4] This low yield and variability in the diagnostic yield of ICA for the detection of obstructive CAD have resulted in the need for first-line gatekeeper tools. Coronary computed tomographic angiography (CCTA) is a non-invasive diagnostic tool that can exclude CAD with a negative predictive value well in excess of 90%. [5–7] As such, CCTA has emerged as a potential gatekeeper, demonstrating that patients undergoing CCTA prior to ICA are up to three times less likely to have normal coronary arteries and are more likely to have obstructive CAD. [8,9] As a result, National Institute for Health and Clinical Excellence (NICE) guidelines have recommended a CCTA-only assessment of patients with atypical or typical angina. [10] However, the application of CCTA to all patients in this manner has been postulated to result in a positive predictive rate for obstructive CAD of only 21% in patients with a positive CCTA who undergo downstream ICA. [11] Multiple risk scores have also been developed and are widely used to systematize risk assessment based on clinical history. This has been guideline-recommended in the evaluation of CAD. [12, 13] Amongst these risk scores is the CAD consortium clinical score (CAD2). [14]

The recent Coronary Computed Tomographic Angiography for Selective Cardiac Catheterization (CONSERVE) trial compared a direct ICA referral strategy to a selective one using CCTA as a gatekeeper. [15] In this study, CCTA reduced ICA normalcy rates by almost 2.5 times compared to the direct ICA arm, suggesting that CCTA can be used to enrich the diagnostic yield of ICA in the detection of obstructive CAD. Of further note, the CCTA group showed a 28% reduction in revascularization.

The advent of Machine Learning (ML) has enabled the autonomous acquisition of knowledge by pattern extraction from large data sets. [16] ML proposes a set of novel algorithms for the construction of inferential and predictive data-driven models, and has been used to predict a variety of cardiovascular outcomes. [17,18]

In this exploratory analysis, we develop and evaluate a ML algorithm to predict obstructive CAD, and compare this algorithm to the CAD2. We also utilize a ML prediction model to gain insight into the relative importance of imaging and clinical variables for revascularization and further compare the effect of choice of imaging modality, using the CONSERVE cohort.

## Methods

### Study design and population

**Fig 1:**
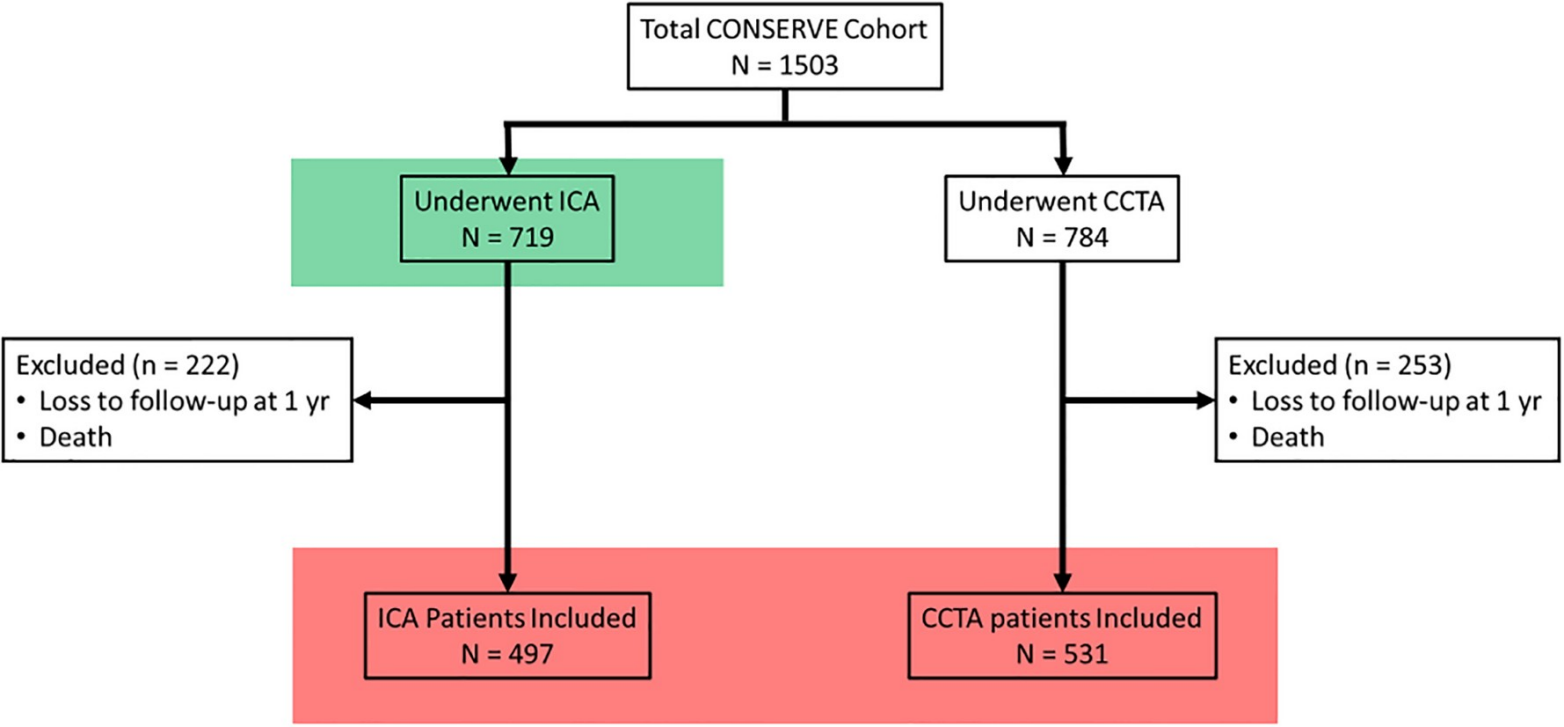
Patient selection. The cohort for revascularization analysis is in the red box, and the cohort for obstructive CAD prediction is in the green box. Abbreviations: CAD = coronary artery disease, CCTA = Coronary computed tomographic angiography, ICA = Invasive Coronary Angiography.

The study design and population have previously been described in detail. [15] In brief, this was a 1:1 randomized, controlled, open-label, international, multicenter trial. Participants were stable patients with suspected but no known CAD referred for nonemergent ICA based upon American College of Cardiology/American Heart Association (ACC/AHA) guidelines for ICA. [19] The original study protocol was approved at each enrolling site by the local institutional review board or ethics committee, and this secondary analysis was reviewed and declared IRB exempt by the institutional review board of Weill Cornell Medicine (statistical and data coordinating center). A selective referral strategy was defined by initial use of CCTA, with ICA performed at the discretion of the local physician informed by the CCTA findings. A direct referral strategy was defined as direct implementation of ICA as otherwise planned before study enrollment. Randomization was performed with 1:1 allocation to the selective referral or direct referral groups. A total of 1,028 patients from 1,503 in the CONSERVE study met eligibility criteria for revascularization analysis. Those that were excluded were due to loss to follow-up or death within 1 year. Of those included, 531 patients were in the CCTA arm and 497 were in the ICA arm (Table 1). 719 patients in the ICA arm of the original 1503 patients in the CONSERVE study were included for prediction of CAD (Fig 1).

**Table 1:** Baseline characteristics.

Characteristic	Total	CCTA	ICA	p Value
N	1028	531 (51.7)	497 (48.3)	
Age	60.6 ± 11.4	60.0 ± 11.7	61.3 ± 11.1	0.09
Female	462 (44.9)	249 (46.9)	213 (42.9)	0.19
Body Mass Index (kg/m2)	25.5 ± 3.9	25.5 ± 4.0	25.5 ± 3.8	1.00
Race / Ethnicity				0.13
Asian	840 (81.7)	439 (82.7)	401 (80.7)	0.41
White	173 (16.8)	86 (16.2)	87 (17.5)	0.58
African American	12 (1.2)	3 (0.6)	9 (1.8)	0.08
Hispanic	1 (0.1)	1 (0.2)	0 (0.0)	1.00
Unknown	2 (0.2)	2 (0.4)	0 (0.0)	0.50
Risk Factors				
Hypertension	590 (57.4)	295 (55.6)	295 (59.4)	0.22
Dyslipidemia	360 (35.0)	180 (33.9)	180 (36.2)	0.43
Diabetes	243 (23.6)	116 (21.8)	127 (25.6)	0.16
Current Smoker (< = 3 mo)	145 (14.1)	72 (13.6)	73 (14.7)	0.60
Former Smoker (> 3 mo)	187 (18.2)	96 (18.1)	91 (18.3)	0.92
Premature Fx of CAD	80 (7.8)	40 (7.5)	40 (8.0)	0.76
Angina Type				
Typical Angina	306 (29.8)	161 (30.3)	145 (29.2)	0.69
Atypical Angina	434 (42.2)	227 (42.7)	207 (41.6)	0.72
Noncardiac Chest Pain	23 (2.2)	16 (3.0)	7 (1.4)	0.08
Asymptomatic	114 (11.1)	62 (11.7)	52 (10.5)	0.54
Other Symptoms				
Dyspnea	127 (12.4)	57 (10.7)	76 (15.3)	0.03
Palpitations	10 (1.0)	4 (0.8)	6 (1.2)	0.54
Dizziness or syncope	6 (0.6)	3 (0.6)	3 (0.6)	1.00
CAD				
No CAD	301 (29.3)	186 (35.0)	115 (23.1)	<0.01
Nonobstructive CAD	355 (34.5)	181 (34.1)	174 (35.0)	0.75
1-vessel CAD	187 (18.2)	93 (17.5)	94 (18.9)	0.56
2-vessel CAD	99 (9.6)	38 (7.2)	61 (12.3)	<0.01
3-vessel or left main stenosis	85 (8.3)	32 (6.0)	53 (10.7)	<0.01

Abbreviations. CAD = coronary artery disease

### Data collection and image analysis

Data collection was performed prospectively. Baseline data related to demographic characteristics, clinical CAD risk factors, medication use, and angina typicality were recorded at recruitment. Out of a total of 1611 patients at initial randomization, 108 were lost to follow up. Of the remaining 1503 patients, 784 were randomized to the CCTA arm and 719 to the ICA arm and underwent data analysis, with the remainder not receiving the allocated test. Sites were instructed to perform ICA and CCTA in accordance with local site practice and societal guidelines. For both ICA and CCTA, the presence or absence of angiographic stenosis ≥50% was recorded by local site physicians, and the maximum stenosis on a per-patient basis was used to define obstructive CAD. Normal ICAs were considered to be those that demonstrated no stenosis, and non-obstructive CAD was defined as maximal stenosis <50%, calculated using the first ICA that occurred within 1 year of enrollment. Revascularization was defined as any non-emergent performance of percutaneous coronary intervention (PCI) or coronary artery bypass graft (CABG) as guided by the ICA or CCTA results in either arm, within 1 year.

### Prediction of significant CAD

ML and CAD2 were used to calculate the AUC for CAD prediction. The CAD2 model requires age, sex, symptoms (typical vs atypical angina), diabetes, hypertension, smoking, hyperlipidemia. [14] CAD2 is guideline-recommended and has been shown to provide best discrimination for the detection of obstructive CAD compared to other existing models. [12, 20]

### Statistical methods

All statistical analyses were performed in R, version 3.5.0. Continuous variables are expressed as mean ± standard deviation, while categorical variables are presented as absolute values and proportions. Continuous variables with normal distribution were compared using Student’s t-test, and categorical variables were compared using chi-square tests. Patients who were lost to follow-up or died within 1 year were censored for 1-year revascularization prediction. The data was randomly split into 80% training set and 20% test set for model building and validation, respectively. Models for baseline obstructive CAD and 1-year revascularization were constructed using extreme gradient boosting (XGBoost) in the training set with 5-fold cross-validation and were tested in the remaining test dataset. [21] XGBoost analyses were based on 91 demographic, clinical, and imaging features. Classification performance was scored with the Area Under the receiver-operation Curve (AUC). A p value <0.05 was considered significant for all analyses.

## Results

Mean age was 60.6 ± 11.5 and 64.3% were female. Mean body mass index (BMI) was 25.5 ± 3.9 kg/m2. Prevalence of diabetes, hypertension, and hyperlipidemia were 23.6%, 57.4%, and 35.0% respectively. 29.8% experienced typical angina, 42.2% had atypical angina, 2.2% had noncardiac chest pain and 11.1% were asymptomatic. The ICA arm had a higher prevalence of 2- and 3-vessel or left main obstructive CAD, and the CCTA arm had a higher prevalence of no CAD. 27.0% and 5.3% of patients underwent downstream noninvasive testing in the ICA and CCTA arms respectively. Revascularization occurred in 18% of the ICA arm versus 13% in the CCTA arm (P = 0.007). This was attributed to a higher PCI rate in the ICA arm of 15% versus 11% in the CCTA arm (P <0.0001). There were no further significant differences between the ICA and CCTA group. 80% of this cohort (822 patients) were randomly selected for algorithm training, and the remaining 20% (206 patients) for validation.

**Fig 2:**
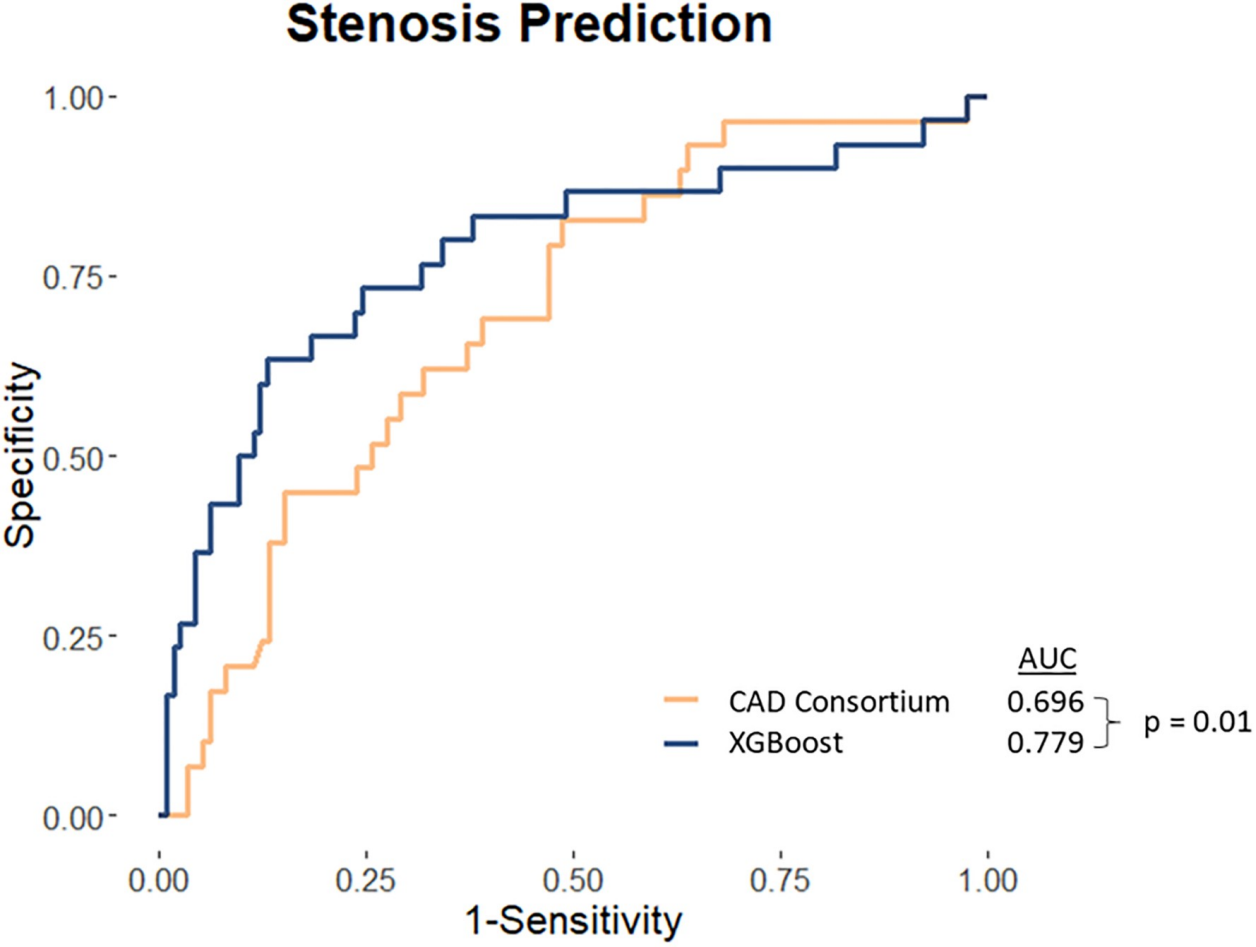
Receiver Operating Characteristics (ROC) analysis for the prediction of obstructive coronary artery disease using non-imaging variables. Abbreviations: AUC = Area under curve, CAD = Coronary artery disease.

**Fig 3:**
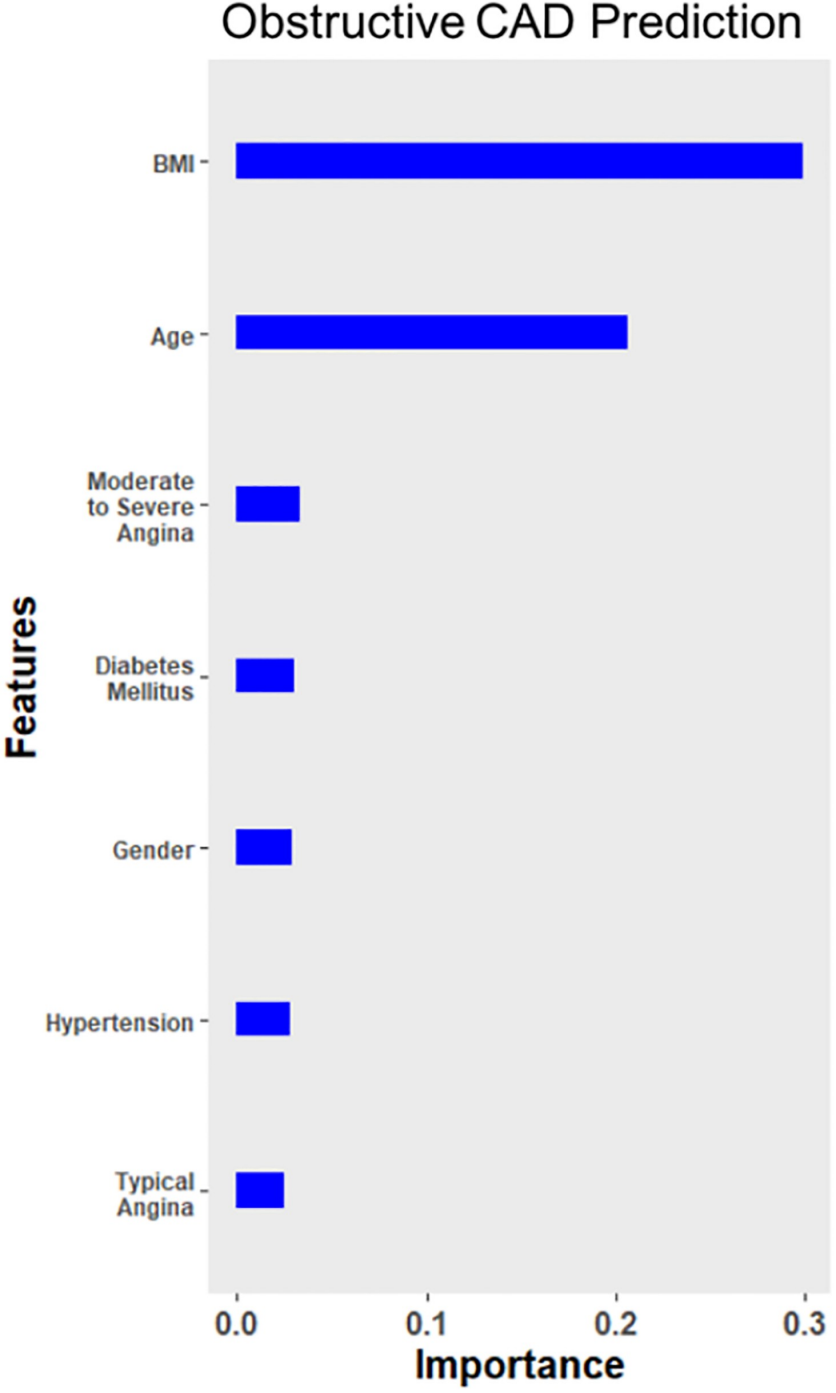
The relative importance of clinical variables in the developed machine learning–based model for the prediction of obstructive coronary artery disease. Abbreviations: BMI = body mass index, CAD = coronary artery disease.

A total of 91 variables were used for the ML models (S1 Table, S1 Fig). For the prediction of obstructive CAD, continuous ROC analysis revealed the AUC for ML model to be 0.779 (95% CI = 0.672–0.886), and CAD2 to be AUC of 0.696 (95% CI = 0.594–0.798). ML exhibited significantly higher AUC compared to CAD2 for this population (p = 0.01) (Fig 2). The ML model was able to achieve an ICA normalcy rate, defined as no obstructive CAD upon imaging study, of 36.7% (11 of 30 patients), and a false negative rate of 13.2% (15 of 114 patients). Additionally, at a probability cutoff of 0.5, ML model achieved a sensitivity and specificity of 86.8% and 63.3%, respectively. The top 7 features are shown in Fig 3. BMI, age, and angina severity were the three most important variables in the prediction of obstructive CAD. With a cutoff set to 0.5, CAD2 had an ICA normalcy rate of 3.4% (1 of 29 patients), a false negative rate of 82.3% (93 of 113 patients), and sensitivity and specificity of 17.7% and 96.6%, respectively.

**Fig 4:**
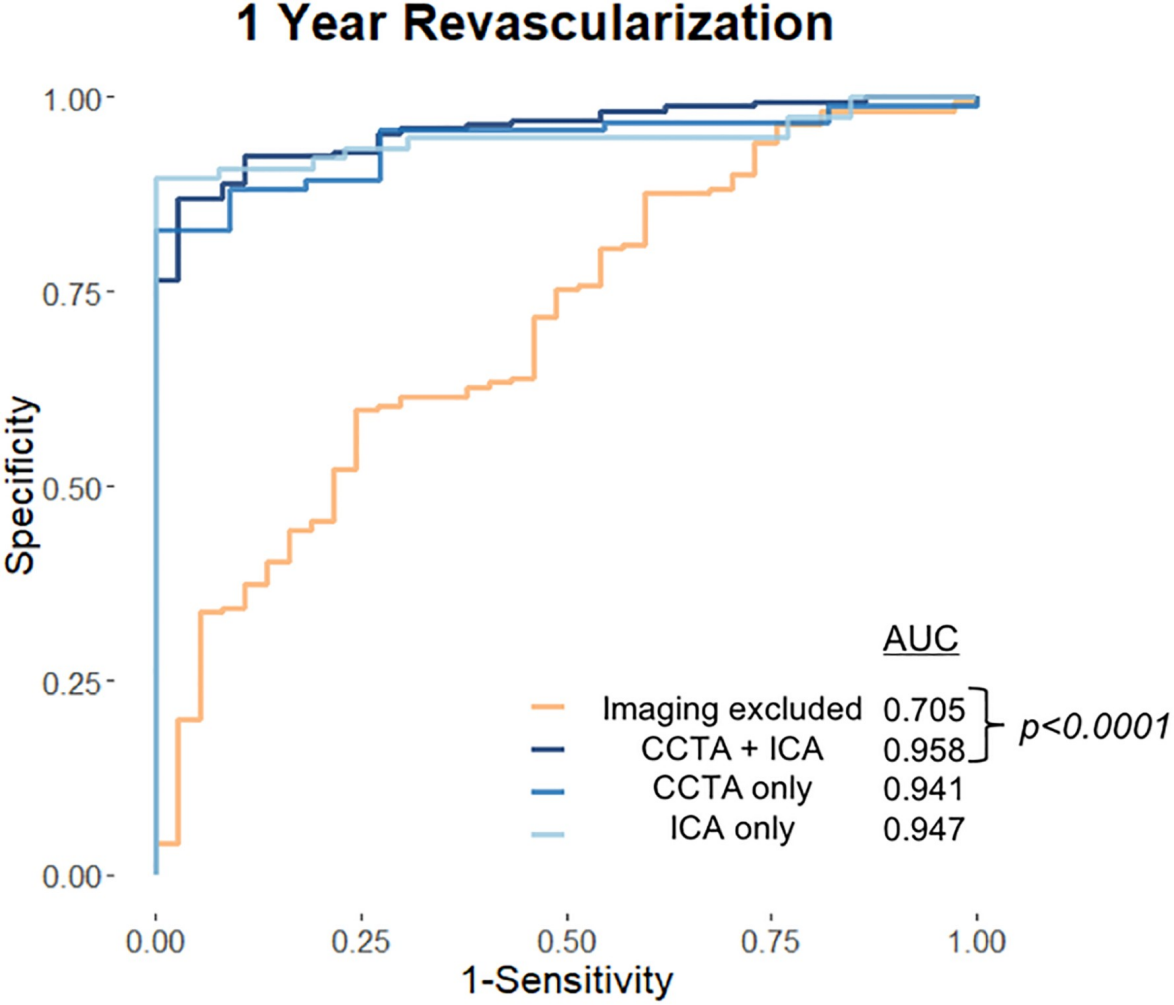
Receiver Operating Characteristics (ROC) analysis for the prediction of 1-year revascularization using non-imaging variables only (orange) and incorporating imaging variables (blue shades). Abbreviations: AUC = Area under curve, CCTA = Coronary computed tomographic angiography, ICA = Invasive coronary angiography.

**Fig 5:**
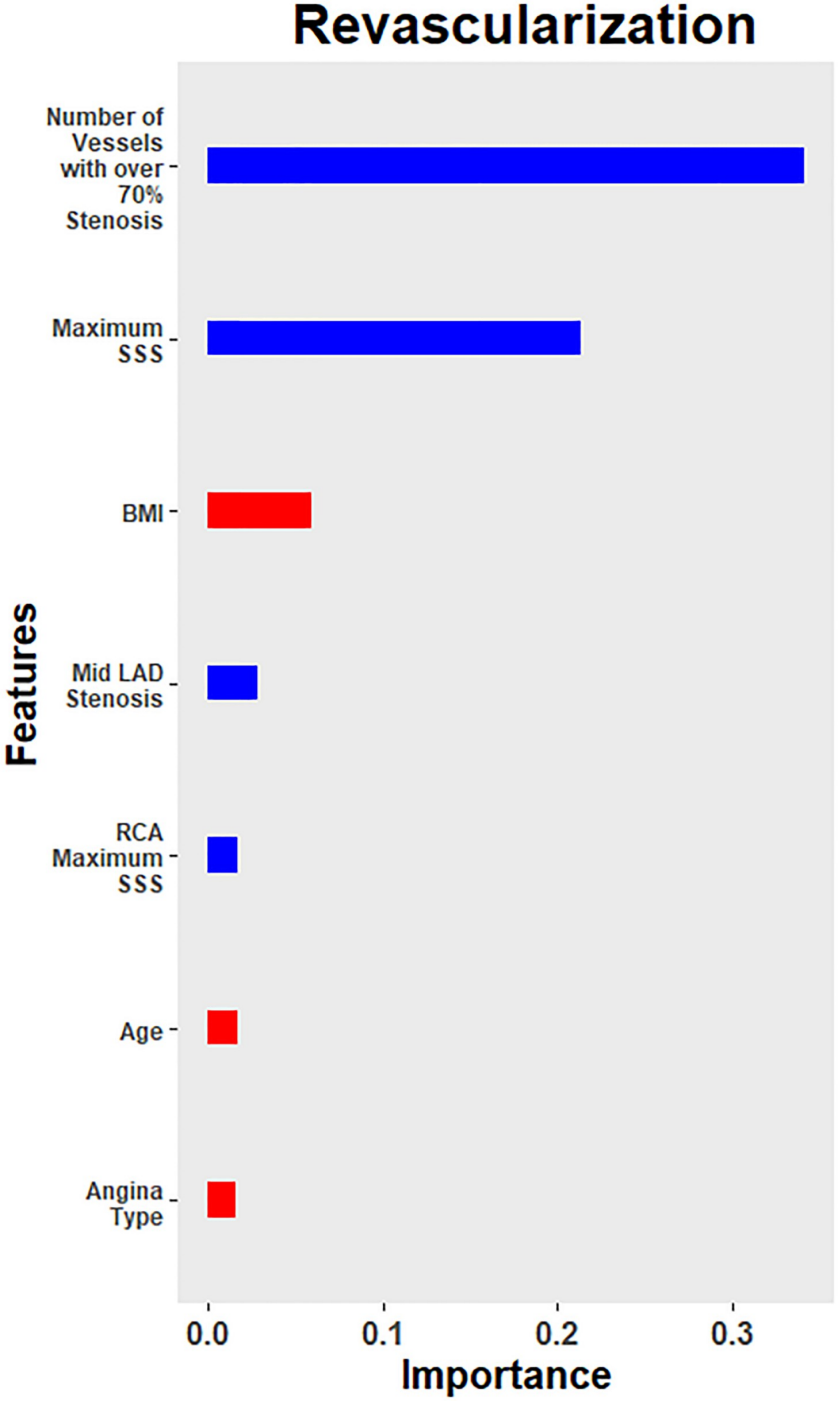
The relative importance of clinical (red) and image-based (blue) variables in the developed machine learning–based model for the prediction of 1-year revascularization. Abbreviations: BMI = body mass index, LAD = left anterior descending coronary artery, RCA = right coronary artery, SSS = maximum segment stenosis severity.

For 1-year revascularization, the ML model obtained an overall AUC of 0.958 (95% CI = 0.933–0.983) (Fig 4). The discriminatory performance of this model did not differ whether the imaging parameters used were from ICA (AUC 0.947, 95% CI = 0.903–0.990) or CCTA (AUC 0.941, 95% CI = 0.895–0.988) (P = 0.90). Overall, with a probability cutoff set to 0.5, the model demonstrated a sensitivity and specificity of 89.2% and 92.9%, respectively. Fig 5 shows the feature importance of the top 7 features, after training with the entire training data set. The top three contributory variables in descending order for the ML prediction model for 1 year revascularization were number of vessels with ≥70% stenosis, maximum segment stenosis severity (SSS), and patient BMI. However, when imaging variables were excluded and only demographics and risk factors were used, the discriminatory performance of the ML prediction model deteriorated, with a decrease in AUC 0.705 (95% CI 0.614–0.795) (P <0.0001) (Fig 4). Excluding imaging variables, the top three contributory features were BMI, age, and angina type (Fig 5).

## Discussion

In this analysis, ML models were developed to predict obstructive CAD, and to gain insight into the role of clinical and imaging variables in the determination of revascularization. The model was also able to predict obstructive CAD with moderate discriminatory performance. BMI was the most important non-imaging feature for both the prediction of obstructive CAD and revascularization, a variable that has not been emphasized in many prior studies.

For the prediction of obstructive CAD, BMI, age, and angina severity were the three clinical features that were the most important contributors. The variables used for this ML prediction model are easily obtainable in the clinic/office setting, i.e. before performing any diagnostic tests. and were selected to be comparable to the preexisting CAD prediction scores. The original Diamond-Forrester model identified age, gender, and angina typicality as main predictors of obstructive CAD. [22] Since then, newer iterations of other models such as CAD2 have suggested additional clinical features (e.g., dyslipidemia, family history, diabetes, current smoker) that contribute to predicting obstructive CAD. [11,14, 23] CAD2, built upon the original CAD consortium basic model to include clinical features, and has been shown to achieve better goodness of fit and discrimination scores compared to other models. [20] In this population, ML model was able to outperform CAD2 in terms of AUC while offering a better balance of false positive and false negative. While CAD2 was able to achieve a much lower false positive rate (3.4% vs 36.7%), it had a significantly higher false negative rate (82.3% vs 13.2%). This suggests that many patients might forego imaging tests under CAD2 risk score due to high false negative rate. Important features noted in this study, as well as those included in many previous models for CAD, are non-invasive and affordable, making it attractive to continually improve screening tools for identifying patients with CAD.

Despite differences in study cohorts, these risk factors are largely concordant with those included in the current model. In a prior external validation of the updated Diamond-Forrester model amongst 3903 patients, chest pain symptoms and sex were the main predictors of obstructive CAD. [24] However, the current ML model suggests a more diminished role for those variables. Instead, BMI is a major contributor to the pre-test probability of obstructive CAD. One possible explanation for this discrepancy could be due to this study’s population, where over 65% of patients were recruited from Korea. [15] The BMI for this population has consistently been shown to be lower than most other geographical regions. [25] This may influence any inference with regards to the role of BMI in this analysis. A longitudinal population study amongst 2,611,540 Korean men and women showed that an increase in BMI was associated with an increase in coronary heart disease, similar to other geographical cohorts, external validation amongst more diverse cohorts is required to ensure applicability and utility. [26] However, the ML model’s improved AUC over other that of other models when applied to this cohort suggests that there could be a need for the development of CAD models for different patient demographics. Currently, BMI is absent in most major CAD risk calculators, but some recent studies have recognized obesity as an important feature contributing to the prediction of CAD. [27–31] As it is a simple variable to measure in an office setting, and is often obtainable from Electronic Health Records (EHR), it is an attractive candidate variable to include in future iterations of CAD risk calculators.

Additional non-invasive variables, including lifestyle factors, have been suggested to influence the risk of CAD which were not available in our dataset. A few studies have reported the importance of work-related features such as office location and shift work, as well as ECG readings and other non-invasive imaging modality variables such as from echocardiograms in the improvement of the prediction of CAD. [30–32] In these studies, using a variety of data mining methods, AUCs of 0.65–0.92 were obtained. In these studies, BMI was not a high-ranked feature, possibly due to the presence of additional features not present in the current study, such as electrocardiogram and additional clinical examination features.

The ICA normalcy rate using the ML model was 36.7% compared to the CCTA (21.1%) or direct ICA (61.5%) arm of the CONSERVE study. [15] Another study showed that 23.8% of the CCTA group and 71.2% of the direct ICA group experienced non-actionable ICA. [33] These results suggest that by drawing associations between multidimensional variables, ML could enhance the gatekeeper function of CCTA and enrich ICA yield further. Moving forward, ML models will likely incorporate imaging results from CCTA, which will further strengthen its role in evaluating risk in patients suspected of obstructive CAD. As data acquisition becomes more multidimensional, the availability of a large amount of information could be integrated to finetune a more precise predictive model for obstructive CAD.

For revascularization, this model incorporated clinical variables easily identified in a patient’s medical history as well as imaging results. Our model suggests that SSS and number of vessels with ≥ 70% stenosis, followed by BMI, were features that strongly contribute to 1 year revascularization. The imaging variables suggest that both lesion-specific stenosis as well as overall plaque burden play a role in revascularization. The contributory role of overall plaque burden towards revascularization has previously been reported. In an international, multicenter prospective observational registry of 1345 patients, the addition of imaging measures of overall plaque burden improved event prediction, mainly comprising revascularization, from and AUC of 0.581 to 0.687. [34] The discriminatory performance of the ML model did not vary when the source of the image-based variables was switched between ICA or CCTA. However, this performance markedly dropped when imaging-based variables were removed.

Whilst the former two imaging variables are intuitively concordant with decision-making for revascularization, the contributory role of BMI is less well recognized. In prior studies, BMI was associated with differential rates of revascularization. [35, 36] In those studies, although BMI was initially associated with increased revascularization rates, this reduced after a certain threshold, and varied according to method of revascularization and coronary anatomy. In this current model, feature importance is based on the gain of each variable, i.e. the relative contribution of the corresponding variable to the model calculated by taking each variable’s contribution for each tree in the model. [37] A higher value of this metric when compared to another variable implies it is more important. Additionally, BMI features vary frequently in the various nodes in the current model. To explore BMI in more depth, a less dimensional, more constrictive regression model could be constructed. However, the current analysis and model is based on the a priori assumption that the relationships between BMI, other variables and revascularization or obstructive CAD are nonlinear. Rather than a simplistic positive association, it is likely multidimensional and complex. Although it bears further investigation, deeper exploration into the relationship between BMI and outcomes using a more targeted modelling approach is outside the scope of this current paper.

A prior ML analysis of 1980 patients predicted early revascularization using single-photon emission computed tomography (SPECT) myocardial perfusion imaging. [38] In that study, functional imaging parameters were used, including perfusion and stress ECG findings, for a total of 55 variables. Concordant to the current analysis, BMI was also found to be the most important non-imaging variable. In the SPECT study, imaging variables contributed the most to the implementation of revascularization, concordant to the current study. In addition to that study, the marked deterioration in our current ML model’s ability after the removal of imaging variables emphasizes the need for imaging over and above traditional non-imaging risk factors. This is also congruent with another prior study on 15207 patients, that showed the AUC for prediction of revascularization drop from 0.91 to 0.63 when switching from CCTA-defined CAD to non-imaging variables. [39]

This model also illustrated that altering the imaging modality from ICA to CCTA did not result in a significant difference in discriminative ability for revascularization. In the SCOT-HEART (Scottish Computed Tomography of the Heart) trial of 4146 patients with stable chest pain CCTA was associated with an apparent increase in coronary revascularization when compared to ICA (11.2% vs 9.7%), although this fell just short of statistical significance (P = 0.061). [40] Similarly and more significantly, the PROMISE (Prospective Multicenter Imaging Study for Evaluation of Chest Pain) trial of 10003 patients saw an almost twofold increase in revascularization in the CCTA arm compared to the ICA arm. [9] The current findings may be in contradiction to the former two trials. This may be reflective of the CONSERVE study that forms its basis, that showed a lower revascularization rate for CCTA (13%) compared to ICA (18%). [15] This may present CCTA as a non-invasive alternative to ICA as a gatekeeper to revascularization, as suggested by other studies. [39, 41]

This ML analysis was intended to be exploratory and hypothesis-generating, and there are several additional limitations of note. The model for revascularization was limited to the confines of the CONSERVE study design, and the emphasis of the revascularization model was to gain insight into the variables most associated with revascularization, rather than to imply a causal relationship. These associations may not carry inferential import in real-world practice. The role of BMI is not necessarily an incremental one, and this analysis did not have the granularity of information required to draw more definite conclusions in this regard. Furthermore, variables known to influence revascularization, such as education level, geographical, local site practice and hospital characteristics, accessibility to angiography, cardiologist in charge and day of admission, were not available. Functional measurements, such as fractional flow reserve (FFR), were not performed. This is because the original aim of the CONSERVE study did not necessitate it. This limitation allows scope for further analysis in other cohorts that have measured FFR. The influence of other unmeasured confounders cannot be ruled out. The relatively small sample of participants and referral bias result in a highly selective patient population. These factors may limit the generalizability of results. More detailed analysis in a larger study may help further identify factors that influence revascularization or predict obstructive CAD.

In conclusion, for the prediction of obstructive CAD, a ML model exhibited comparable performance to prior history-based scores, but further external validation is needed. This ML analysis showed BMI to be an important variable, although it is currently not included in most risk scores. Imaging variables were the most associated with 1 year revascularization, and imaging modality did not influence the model performance. Furthermore, removal of imaging variables reduced model performance significantly. This analysis provides a basis for further ML exploration into the role of factors that both influence predict obstructive CAD as well as affect revascularization.

## Supporting information

S1 Data (XLSX)

S1 Table Input machine learning variables. (DOCX)

S1 Fig Sample model decision trees. BMI (red) features frequently in numerous nodes in the decision tree for the XGBoost model. The model involves hundreds of such trees. Abbreviations: BMI = body mass index.
(DOCX)
