# Supplementary material for: Machine learning insight into the role of imaging and clinical variables for the prediction of obstructive coronary artery disease and revascularization: An exploratory analysis of the CONSERVE study
Source: PLoS One. 2020 Jun 25;15(6):e0233791. doi: 10.1371/journal.pone.0233791 (PMC7316297; doi:10.1371/journal.pone.0233791)
Supplement: S1 Table — (DOCX) [file pone.0233791.s002.docx]

| **Supplemental Table 1. Input Machine Learning Variables** |
| --- |
| [1] "Subject_age" |
| [2] "Gender__Female" |
| [3] "Gender__Male" |
| [4] "race_ethnicity_African American" |
| [5] "race_ethnicity_Asian" |
| [6] "race_ethnicity_Hispanic" |
| [7] "race_ethnicity_Unknown" |
| [8] "race_ethnicity_White" |
| [9] "bmi" |
| [10] "bl_anginatype_Asymptomatic" |
| [11] "bl_anginatype_Atypical angina" |
| [12] "bl_anginatype_Non-cardiac pain" |
| [13] "bl_anginatype_Other" |
| [14] "bl_anginatype_Typical or unstable angina" |
| [15] "bl_cardrisk_currentsmoker_0" |
| [16] "bl_cardrisk_currentsmoker_1" |
| [17] "bl_cardrisk_formersmoker_0" |
| [18] "bl_cardrisk_formersmoker_1" |
| [19] "bl_cardrisk_dm_0" |
| [20] "bl_cardrisk_dm_1" |
| [21] "bl_cardrisk_fxcad_0" |
| [22] "bl_cardrisk_fxcad_1" |
| [23] "bl_cardrisk_hlp_0" |
| [24] "bl_cardrisk_hlp_1" |
| [25] "bl_cardrisk_htn_0" |
| [26] "bl_cardrisk_htn_1" |
| [27] "bl_nist_results_Asymptomatic +2 Risk Factors (RF) + Abnormal NIST" |
| [28] "nvd_50" |
| [29] "bl_nist_results_Recurrent hospitalization for Chest Pain + Abnormal/equivocal NIST" |
| [30] "Max_sss" |
| [31] "bl_nist_results_Symptomatic (chest pain) + Abnormal NIST" |
| [32] "LCx_max_sss" |
| [33] "bl_nist_results_Symptomatic (chest pain) + Abnormal NIST ___Recurrent hospitalization for Chest Pain + Abnormal/equivocal NIST" |
| [34] "bl_nist_results_Symptomatic (chest pain) + Abnormal NIST_Asymptomatic +2 Risk Factors (RF) + Abnormal NIST" |
| [35] "bl_nist_results_Symptomatic (chest pain) + Abnormal NIST_Worsening NIST" |
| [36] "bl_nist_results_Worsening NIST" |
| [37] "bl_nist_results_Worsening NIST __Recurrent hospitalization for Chest Pain + Abnormal/equivocal NIST" |
| [38] "bl_nist_worsening_0" |
| [39] "bl_nist_worsening_1" |
| [40] "bl_nist_recurrent_0" |
| [41] "bl_nist_recurrent_1" |
| [42] "bl_nist_symptom_0" |
| [43] "bl_nist_symptom_1" |
| [44] "bl_nist_asymptom_0" |
| [45] "bl_nist_asymptom_1" |
| [46] "bl_nist_0" |
| [47] "bl_nist_1" |
| [48] "accaha_nist_0" |
| [49] "accaha_nist_1" |
| [50] "prior_othernist_0" |
| [51] "prior_othernist_1" |
| [52] "prior_anynist_0" |
| [53] "prior_anynist_1" |
| [54] "abnormnist_0" |
| [55] "abnormnist_1" |
| [56] "blsymptoms_Any angina, not evaluable by non-invasive stress testing" |
| [57] "blsymptoms_Heart failure with normal ejection fraction of unknown e" |
| [58] "blsymptoms_Mild or moderate angina, which is intolerant to medical" |
| [59] "blsymptoms_Moderate or severe angina, which improves to mild to moderate" |
| [60] "bl_Angina_Type_arrhythmia" |
| [61] "bl_Angina_Type_Asymptomatic" |
| [62] "bl_Angina_Type_Atypical" |
| [63] "LAD_max_sss" |
| [64] "bl_Angina_Type_Dizziness" |
| [65] "bl_Angina_Type_Dyspnea" |
| [66] "bl_Angina_Type_effort" |
| [67] "bl_Angina_Type_Non-cardiac" |
| [68] "RCA_max_sss" |
| [69] "bl_Angina_Type_None" |
| [70] "bl_Angina_Type_palpitation" |
| [71] "SIS" |
| [72] "bl_Angina_Type_syncope" |
| [73] "Severity....Diag.2....Severity" |
| [74] "Severity....Diag.1....Severity" |
| [75] "Severity....R.PLB....Severity" |
| [76] "bl_Angina_Type_vasospatic" |
| [77] "Severity....Prox.RCA....Severity" |
| [78] "Severity....Mid.RCA....Severity" |
| [79] "Severity....Distal.RCA....Severity" |
| [80] "Severity....LM....Severity" |
| [81] "Severity....Prox.LAD....Severity" |
| [82] "Severity....Mid.LAD....Severity" |
| [83] "Severity....Distal.LAD....Severity" |
| [84] "Severity....Ramus....Severity" |
| [85] "Severity....Prox.Circ....Severity" |
| [86] "Severity....Distal.Circ....Severity" |
| [87] "Severity....OM1....Severity" |
| [88] "Severity....OM2....Severity" |
| [89] "Severity....L.PDA....Severity" |
| [90] "Severity....R.PDA....Severity" |
| [91] "Severity....L.PLB....Severity" |
