# Supplementary material for: Machine learning insight into the role of imaging and clinical variables for the prediction of obstructive coronary artery disease and revascularization: An exploratory analysis of the CONSERVE study
Source: PLoS One. 2020 Jun 25;15(6):e0233791. doi: 10.1371/journal.pone.0233791 (PMC7316297; doi:10.1371/journal.pone.0233791)
Supplement: S1 Fig — BMI (red) features frequently in numerous nodes in the decision tree for the XGBoost model. The model involves hundreds of such trees. Abbreviations: BMI = body mass index. (DOCX) [file pone.0233791.s003.docx]

**Supplemental Figure 1. Sample Model Decision Trees**


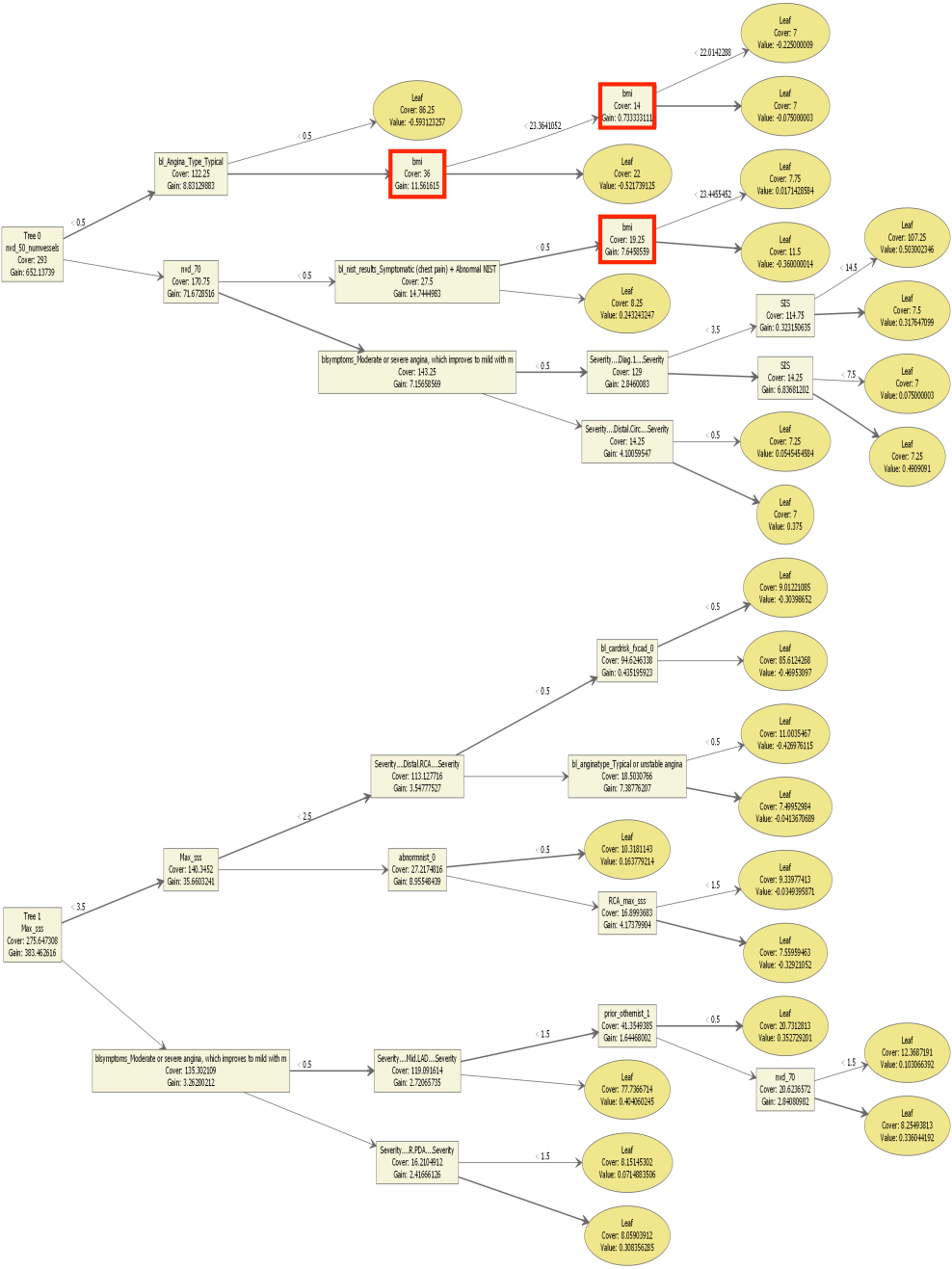


**Supplemental Figure 1:** BMI (red) features frequently in numerous nodes in the decision tree for the XGBoost model. The model involves hundreds of such trees.

Abbreviations: BMI = body mass index.
